# Adherent Self-Renewable Human Embryonic Stem Cell-Derived Neural Stem Cell Line: Functional Engraftment in Experimental Stroke Model

**DOI:** 10.1371/journal.pone.0001644

**Published:** 2008-02-20

**Authors:** Marcel M. Daadi, Anne-Lise Maag, Gary K. Steinberg

**Affiliations:** Department of Neurosurgery and Stanford Stroke Center, Stanford University School of Medicine, Stanford, California, United States of America; University of Sydney, Australia

## Abstract

**Background:**

Human embryonic stem cells (hESCs) offer a virtually unlimited source of neural cells for structural repair in neurological disorders, such as stroke. Neural cells can be derived from hESCs either by direct enrichment, or by isolating specific growth factor-responsive and expandable populations of human neural stem cells (hNSCs). Studies have indicated that the direct enrichment method generates a heterogeneous population of cells that may contain residual undifferentiated stem cells that could lead to tumor formation in vivo.

**Methods/Principal Findings:**

We isolated an expandable and homogenous population of hNSCs (named SD56) from hESCs using a defined media supplemented with epidermal growth factor (EGF), basic fibroblast growth factor (bFGF) and leukemia inhibitory growth factor (LIF). These hNSCs grew as an adherent monolayer culture. They were fully neuralized and uniformly expressed molecular features of NSCs, including nestin, vimentin and radial glial markers. These hNSCs did not express the pluripotency markers Oct4 or Nanog, nor did they express markers for the mesoderm or endoderm lineages. The self-renewal property of the hNSCs was characterized by a predominant symmetrical mode of cell division. The SD56 hNSCs differentiated into neurons, astrocytes and oligodendrocytes throughout multiple passages in vitro, as well as after transplantation. Together, these criteria confirm the definitive NSC identity of the SD56 cell line. Importantly, they exhibited no chromosome abnormalities and did not form tumors after implantation into rat ischemic brains and into naïve nude rat brains and flanks. Furthermore, hNSCs isolated under these conditions migrated toward the ischemia-injured adult brain parenchyma and improved the independent use of the stroke-impaired forelimb two months post-transplantation.

**Conclusions/Significance:**

The SD56 human neural stem cells derived under the reported conditions are stable, do not form tumors in vivo and enable functional recovery after stroke. These properties indicate that this hNSC line may offer a renewable, homogenous source of neural cells that will be valuable for basic and translational research.

## Introduction

To date there have been no effective treatments for improving residual structural and functional deficits resulting from stroke. Current therapeutic approaches, such as the use of thrombolytics, benefit only 1 to 4% of patients [1]. Consequently, the majority of stroke patients experience progression of ischemia associated with debilitating neurological deficits. Recent evidence has suggested that the transplantation of cells derived from cord blood, bone marrow or brain tissue (fetal and adult) enhances sensorimotor function in experimental models of stroke [2], [3]. However, the normal human-derived somatic stem cells used in these studies have a limited capacity to differentiate into the diverse neural cell types optimal for structural and physiological tissue repair and are not amenable for large-scale cell production.

Unlike other sources of stem cells, hESC lines possess a nearly unlimited self-renewal capacity and the developmental potential to differentiate into virtually any cell type of the organism. As such, they constitute an ideal source of cells for regenerative medicine. The successful derivation of hESC lines from the inner cell mass of preimplantation embryos and their long-term maintenance in vitro over multiple passages has been demonstrated [4] and standardized. Differentiation and enrichment processes that direct hESCs towards a neural lineage have also been achieved. To promote neuralization, ESCs were cultured in a defined media supplemented with morphogens or growth factors [5], [6], [7] or cultured under conditions that promote “rosettes”, structures morphologically similar to the developing neural tube [8], [9]. This neuralization process has proven invaluable in understanding the specification of hESC-derived neural tissue [10], [11], [12]. However, the enriched neural progeny derived displayed overgrowth and limited migration after grafting into normal newborn mice [13] and lesioned adult rat striatum [12], [14], [15], [16]. The inner cores of these grafts contained tumorigenic precursor cells (reviewed in [17]). These findings suggest that neural cells generated by acute exposure to growth factors and/or morphogens may still be heterogeneous and potentially tumorigenic.

We report an alternative method for the isolation and the perpetuation of a multipotent hNSC line from the hESCs with a primitive mode of self-renewal. We also demonstrate their long-term expansion, non-tumorigenic properties and functional engraftability in an experimental model of stroke.

## Results

### 1. In vitro isolation, growth and differentiation of hESC-derived hNSCs

**Figure 1:**
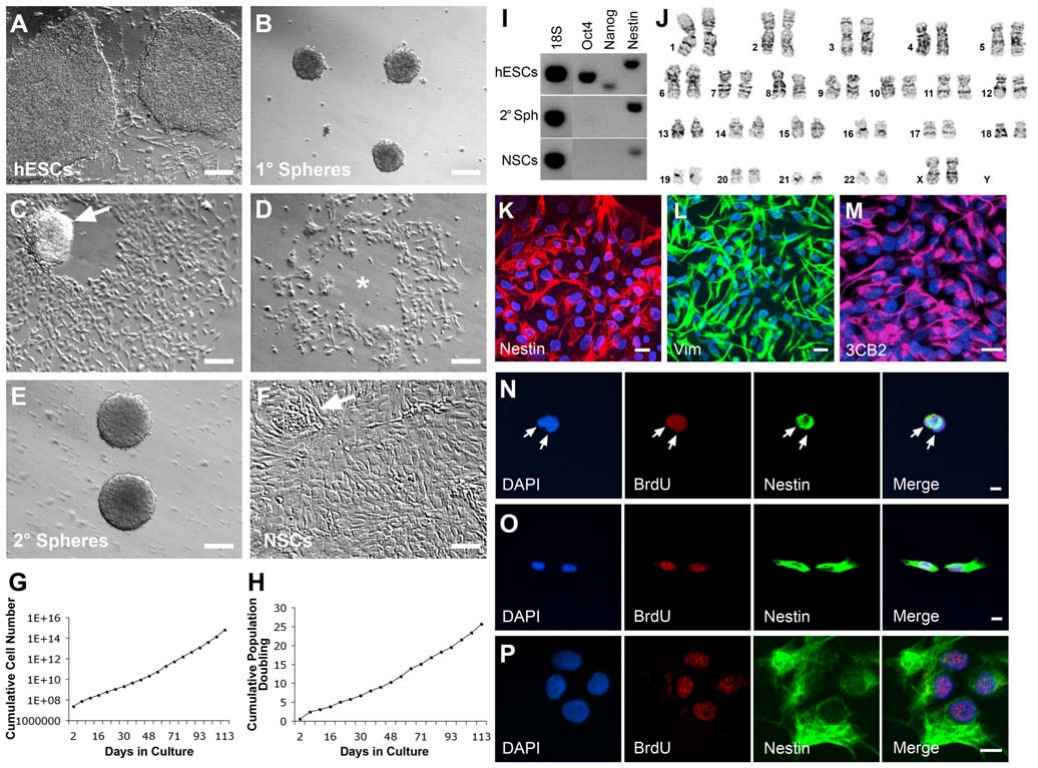
Isolation and purification of hNSCs from the hESCs. The hESCs were grown on a mouse feeder layer (A). Primary neurospheres (B) were isolated and replated to eliminate other non-neural cells. The selectively harvested secondary neurospheres (arrow in C), left behind hollow cores in the surface area (star in D) where they attached earlier. They were perpetuated for an additional 5 passages (E). These 2° spheres were then passaged using a single cell dissociation protocol (F). Arrow in F shows an example of a focus of proliferating cells. (G, H) The hNSCs were passaged every 5–7 days, as described in the Methods section. Starting from an initial population of 1 million cells, the cumulative cell number was calculated at each passage as the fold of increase×the total cell number and plotted as logarithm with base 2 in function of time (G). The cell perpetuation (G) and population doubling (H) analysis demonstrated the continuous and stable growth of the hNSCs. (I) RT-PCR analysis showing the down regulation of the pluripotency transcripts Oct4 and Nanog in secondary neurospheres and in expanded hNSCs at passage 8 (P8). (J) Cytogenetic evaluation of the SD56 hNSCs line at passage 12 by standard G-banding was performed. Twenty metaphase cells were analyzed and showed a normal female chromosome complement (46,XX). Isolated and expanded hNSCs expressed the neural precursor cell markers nestin (K), Vimentin (L) and the radial glial cell marker 3CB2 (M) in virtually all the progeny. (N-P) Clonal self-renewal ability of the isolated hNSCs through symmetrical cell division. Single (N), two-cell stage (O) and four-cell stage (P) of a hNSC proliferating over a 3-day culture period. Note the symmetrical segregation of BrdU and nestin in the progeny. Bars: (A, B, C, D) 200 µm; (E, F) 100 µm; (K–M) 20 µm; (N–P) 10 µm.

The hESCs were maintained and expanded on mouse feeder layer in media supplemented with bFGF (Figure 1A). After cell dissociation, a portion of the hESCs was cultured in serum free medium containing EGF, bFGF and LIF. These factors are known to stimulate the proliferation of human fetal-derived NSCs [18], [19]. After 3 days in vitro (DIV), there was selective survival and growth of cells that aggregated in clusters or spheres (Figure 1B). These primary spheres were harvested and replated in fresh media. During the following week, the spheres attached to the flask and a fibroblast-like cell population began to migrate out (Figure 1C). Secondary spheres (2° spheres) were generated from these cultures and lifted off by the end of the week leaving a hollow in the middle of the attached cells (Figure 1D). The floating 2° spheres were collected and replated in fresh growth medium for 2 weeks. The cultures were then passaged by collagenase cell dissociation every 7 DIV for an additional 4 passages (Figure S1). At the 5^th^ and 6^th^ passages, spheres were dissociated into a single-cell suspension using trypsin-EDTA. At this stage there was a change in the hNSCs' adherent properties, and the cells began to grow as a monolayer with multiple foci of cells throughout the culture (Figure 1F). The adherent hNSC culture stained uniformly for nestin (Figure 1K), vimentin (Figure 1L) and with the radial glial marker 3CB2 (Figure 1M) indicating their homogeneity and NSC identity. Under these culture conditions, it is noteworthy that we did not observe the formation of rosettes which has been previously reported to occur under certain conditions during neuralization of hESCs [8], [20], [21]. RT-PCR analysis confirmed that these hNSCs did not express the pluripotency transcripts Oct-4 and Nanog (Figure 1I). Furthermore, the hNSCs did not express transcripts for *brachyury* and *foxa2,* marker genes for mesoderm and endoderm respectively (negative result, data not shown).

To ascertain self-renewal ability under clonal conditions, a single cell suspension was plated at clonal density (1–2 cell/10 µl). To determine if the hNSCs divide symmetrically, we pulsed cultures with the thymidine analog, bromodeoxyuridine (BrdU), after plating and looked for the expression of nestin in the progeny. Cultures were fixed after 1, 2 or 3 DIV (Figure 1N–P). After 2 days, plated single cells first underwent a symmetric cell division and gave rise to daughter cells that were both positive for BrdU and nestin. The clone of cells continued to grow over the 3 DIV and all the progeny expressed nestin. BrdU labeling demonstrated that it was rare for only one daughter cell to inherit the BrdU and thus had undergone asymmetric segregation of the chromatids (Figure S2). G-band karyotyping of these hNSCs confirmed the normal, non-transformed nature of cells after 12 passages (Figure 1J). We named the derived hNSCs SD56 (intermittently referred to as SD56 hNSCs or hNSCs).

Under these defined growth conditions, the hNSCs showed stable growth with a 2.7±0.2 fold increase every 5 to 7 days (Figure 1G). The population doubling at each passage averaged at 1.4±0.1 (Figure 1H). The viability of hNSCs at each passage was consistent at the approximate value of 98%. The projected cumulative cell numbers demonstrated that trillions of cells could be generated over a period of 5 months (Figure 1G). We expanded the isolated hNSCs lines for over 20 passages with a stable phenotype. An initial cell bank of 75 vials containing 2 to 5 million cells each was generated and cryopreserved.

**Figure 2:**
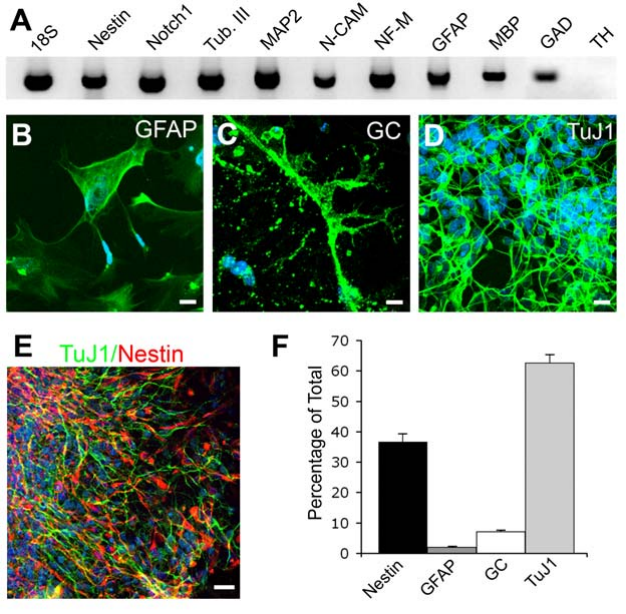
hESC-derived hNSCs spontaneously differentiated into the 3 principal central nervous system cell types. Dissociated hNSCs were washed free of growth factors and plated on poly-L-onithine coated glass coverslips. Differentiated cultures were either harvested after 2 DIV for total RNA extraction and RT-PCR analysis or fixed after 10 DIV and processed for indirect immunocytochemistry. (A) Differentiated hNSCs expressed the neural-specific transcripts nestin and Notch1 as well as transcripts: for neurons (β-tubulin class III, MAP-2, NCAM and medium-size neurofilament, NF-M), for astrocytes (GFAP) and for oligodendrocytes (MBP). The hNSCs expressed transcripts for GAD, but not for TH. B, C & D, morphology of NSC-derived progeny differentiated into GFAP+ astrocytes (B), GC-expressing oligodendrocytes (C) and TuJ1+ neurons (D), DAPI (blue) show life cell nuclei. (E) Photo showing cultures double-immunostained for TuJ1 (green) and nestin (red) (DAPI, blue). (F) Quantitative analysis of immunostained 10 day-old cultures for the 3 neural cell types. Results are mean±s.e.m. of three independent experiments, each performed in duplicate. Bars: (c) 20 µm; (d, e) 10 µm.

Upon removal of the mitogenic factors and plating on a coverslip pre-coated with poly-L-ornithine (PLO) substrate, the hNSCs spontaneously differentiated into neurons, astrocytes and oligodendrocytes, a property that is consistent with normal multipotent hNSCs (Figure 2). After 2 DIV, hNSCs expressed transcripts for the neural-specific genes nestin, Notch1 and neural cell adhesion molecule (N-CAM) (Figure 2A) and for the lineage specific markers β-tubulin class III, medium-size neurofilament (NF-M) and microtubule-associated protein 2 (MAP-2) for neurons, GFAP for astrocytes and myelin basic protein (MBP) for oligodendrocytes (Figure 2A). Transcripts for the GABAergic cell marker glutamic acid decarboxylase (GAD) were expressed, but transcripts for the tyrosine hydroxylase (TH), a marker for dopaminergic neurons, were undetectable. Immunocytochemical analysis (Figure 2B–F) of 10 day-old cultures demonstrated that the proportion of nestin+ cells was 36.6±2.7%, 62.5±2.8% expressed the neuronal marker TuJ1, 1.9±0.3% expressed the astrocytic marker GFAP and 7.1±0.4% were oligodendrocytes and expressed galactocerebrocide (GC) (Figure 2F). A subset (9.8±1.6%) of the GFAP+ astrocytes co-expressed nestin.

### 2. The isolated hNSCs are normal and do not form tumors in normal nude rats

The self-renewal and pluripotent abilities of the hESCs are also associated with tumorigenic properties. Therefore, the first critical step toward developing therapeutic hNSCs is to verify that they are non-tumorigenic. The monolayer culture of SD56 hNSCs clearly demonstrated contact inhibition of growth, a normal karyotype and did not express the pluripotency transcripts Oct-4 and Nanog. Removal of mitogenic factors and continued culture on plastic resulted in cell senescence that is characteristic of non-transformed cells. To determine whether SD56 hNSCs form tumors in vivo, we transplanted them at high density (see Methods) into the forebrain and subcutaneously into the flank of nude rats. The animals were kept for a two-month post-transplant survival period. To label mitotically active cells in vivo during S-phase, the rats were injected IP with BrdU (50 mg/kg) every 8 hours during the last 24 hours before euthanasia. The transit amplifying endogenous precursors located in the subventricular zone (SVZ) were labeled (Figure S3); however, we were unable to detect grafted SD56 hNSCs co-localizing the human-specific nuclear marker hNuc and BrdU (Figure S3). No surviving SD56 hNSCs were detected in the flank of the transplanted animals suggesting that the grafted cells are not tumorigenic.

### 3. Transplanted cells survived, migrated toward and engrafted into the stroke-damaged host tissue

**Figure 3:**
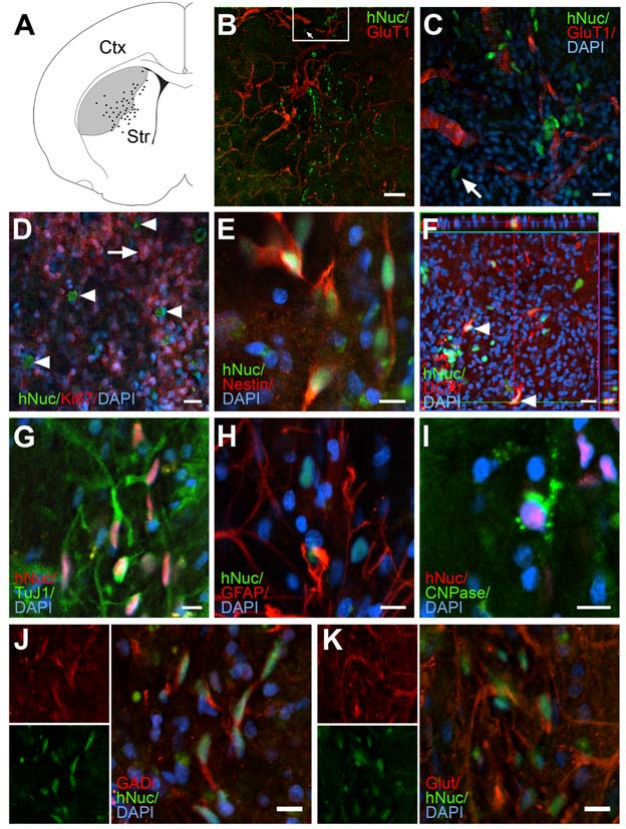
Dispersion, engraftment and differentiation of the hNSCs in stroke-lesioned animals. (A) Schematic drawing of a frontal section through the striatum illustrating the dispersion of grafted hNSCs in the focal ischemia-lesioned parenchyma (shaded area). (B, C) Photos show frontal sections through the graft in the striatum immunostained with the human specific antibodies: anti-hNuc (green in B & C) and anti-GluT1 (red, B & C) showing blood vessels and dispersed hNSCs in the graft zone. C: higher magnification of the inset in B. (D–I) Photos taken from frontal sections through the graft in the striatum double immunoprocessed for cell proliferation and neural lineage markers. (D) Note the endogenous Ki67+ cells (red cells, arrow) in the stroke damaged area and the hNuc+ (green)/Ki67- grafted hNSCs (arrowheads). (E) Examples of grafted SD56 hNSCs showing co-expression of hNuc (green) and nestin (red). (F) Confocal 3D reconstructed orthogonal images of the hNuc+(green)/DCX+(red) NSCs (arrowheads) viewed in the *x-z* plan on the top and *y-z* plan on the right. (G) Examples show the majority of grafted NSC progeny co-expressing hNuc (red) and the neuronal marker TuJ1 (green). Grafted NSCs rarely differentiate into GFAP+ astrocytes (H). In I, rare example of grafted NSC progeny becoming an oligodendrocyte identified by the expression of CNPase (green). Grafted NSCs expressed the GABAergic marker GAD65/67 (J) and rarely expressed glutamate (K). (Abbreviations: Cx: cortex, Str: striatum). Bars: (B, C) 100 µm; (D, F) 20 µm; (E, G–K) 10 µm.

To investigate the survival and functional engraftment in an injury environment, hNSCs (4×10^5^) were transplanted into the ischemic boundary zone in the rat striatum one week after the middle cerebral artery occlusion (MCAO) was performed. Animals were euthanized two months later and the brains processed for histo-pathology and immunocytochemistry. Grafted SD56 hNSCs, identified with hNuc, demonstrated a 37.0±15.8% survival rate and a remarkable dispersion toward the stroke-damaged tissue with no sign of overgrowth or tumorigenesis. The majority of grafted cells (61.2±4.7%) migrated at least 200 µm away from the injection site and penetrated an average distance of 806.4±49.3 µm into the stroke-damaged tissue (Figure 3A–C). Immunostaining with the blood vessel marker, GluT1, revealed dilated vessels in the infarcted striatum and a close association between vessels and the grafted hNSCs (Figure 3B, 3C). The grafted cells rarely expressed the proliferation marker Ki67 (Figure 3D), 29.8±4.4% expressed nestin (Figure 3E), 6.5±0.9% expressed doublecortin (DCX) and 60.8±8.1% were TuJ1+ (Figure 3F, G). Grafted cells rarely co-expressed the astroglial marker GFAP (Figure 3H) or differentiated into CNPase-expressing oligodendrocytes (Figure 3I). Immunostaining for GAD demonstrated that 25.1±2.3% of grafted hNSCs differentiated into GABAergic neurons while less than 2% were positive for glutamate (Figure 3J, K).

### 4. Transplanted cells improve sensorimotor function of the stroke-disabled forelimb

**Figure 4:**
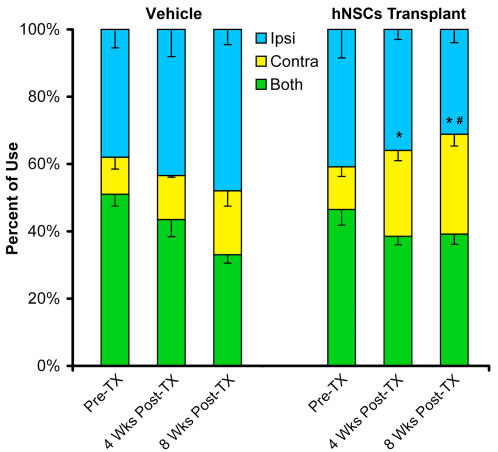
Transplantation of NSCs improves sensorimotor function of the stroke-disabled forelimb. Forelimb use during spontaneous lateral exploration was measured in the cylinder test (see Method and Results sections for details). Groups of vehicle injected (n = 7) and hNSCs (n = 10) transplanted are represented. The animals were tested as described in Method section. Note the significant increase in the independent use of the impaired contralateral forelimb at 4 and 8 weeks post transplantation (*P*<0.05 vs pre-transplant group). The contralateral forelimb remained impaired in the vehicle treated group at 4 and 8 weeks post-injection. Bars represent percentages±s.e.m. of steps taken by the ipsilateral, contralateral and both forelimbs simultaneously. *P<0.05 vs pre-transplant group; ^#^ *P*<0.05 vs vehicle groups.

We asked whether transplanted SD56 hNSCs could enhance the recovery of sensorimotor function that is compromised in the stroke-injured rats. We used the cylinder test to measure the sensorimotor asymmetry in forelimb use during spontaneous exploration [22]. To establish the baseline of the stroke-induced sensorimotor deficit, spontaneous behavior of rats in a transparent cylinder was videotaped one week after stroke (pre-transplant, Figure 4). Tests were then conducted 4 and 8 weeks after vehicle and SD56 hNSCs transplantation. Stable asymmetry in forelimb use was observed 7 days post-stroke (pre-transplant, Figure 4). Ischemic rats used their impaired forelimbs (contralateral to lesion) during lateral exploration less than they did before stroke. Transplantation of SD56 hNSCs significantly enhanced the independent use of the impaired contralateral forelimb 4 weeks post transplantation (P<0.05 vs pre-transplant). Eight weeks after transplantation the improvement in the use of the impaired forelimb was stable and significant when compared to the pre-transplant group and significantly improved in comparison to vehicle treated group at 8 weeks (Figure 4). In the vehicle treated group, the independent use of the contralateral forelimb remained impaired 4 and 8 weeks post-injection. In an independent study and using the same MCAO rat animal model, we found that transplantation of dermal fibroblasts did not improve the stroke-induced motor deficits (unpublished data).

## Discussion

Our results indicate that a self-renewable and homogenous population of hNSCs, SD56, was derived from hESCs using defined media supplemented with a specific combination of growth factors. The SD56 hNSCs grew as an adherent monolayer culture, uniformly expressed molecular features of hNSCs including nestin, vimentin and the radial glial marker 3CB2, and did not express the pluripotency markers Oct4 or Nanog. The self-renewal property of the hNSCs was characterized by a predominant symmetrical mode of cell division. They exhibited no chromosomal abnormalities and demonstrated non-tumorigenic properties after implantation into ischemic brains and into naïve nude rat brains and flanks. Furthermore, the transplanted SD56 hNSCs migrated toward the stroke-damaged adult brain parenchyma, engrafted and improved the independent use of the stroke-impaired forelimb.

Maintenance of stem cells requires symmetrical and asymmetrical cell divisions to both expand and to give rise to specialized progeny of a specific tissue (reviewed in [23]). In vivo, a complex cellular micro-environment or niche ensures the self-maintenance property of NSCs [24], [25], [26], [27]. In vitro, defined growth factors and extracellular matrices support stem cell self-renewal [28], [29]. The embryonic stem cells can propagate in a predominantly proliferative symmetrical mode, leading to homogeneous cell cultures growing relatively quickly with minimal cell differentiation [30], [31], [32], [33], [34]. Tissue specific stem cells, however, self-renew in a predominant asymmetric mode to maintain them selves and compensate for the loss of differentiated cells due to disease or injury. Thus, NSCs isolated from developing or adult brain grow as a mixture of undifferentiated and differentiated cells due the predominant asymmetrical mode of cell division [35], [36], [37], [38], [39], [40], [41]. A recent study has reported that a murine ESC-derived NSC line (LC1) is propagated as an adherent homogenous culture with a dominant mode of symmetrical self-renewal [21]. A combination of EGF and FGF2 was sufficient to propagate these NSCs as an adherent monolayer. However, the SD56 hNSC line described here required the combination of EGF, bFGF and LIF for self-maintenance. Although there are morphological and molecular similarities between our hNSCs and the NSCs previously described [21], the methods of isolation and growth are different. In addition to the different combination of growth factors used, the hNSC line we have isolated did not go through the rosette-structure stage. The in vitro analysis of BrdU incorporation and nestin expression indicated that our hNSCs divide predominantly symmetrically. This type of growth pattern is characteristic of primitive normal stem cells undergoing mostly symmetric cell division to increase the stem cell pool at the early stage of development or during tissue regeneration after injury [23]. RT-PCR and immunocytochemistry analysis demonstrated that these undifferentiated SD56 cells did not express any pluripotency, endodermal or mesodermal markers. Furthermore, the SD56 hNSCs described here exhibited the multipotential characteristic to differentiate into neurons, astrocytes and oligodendrocytes both in vitro and upon transplantation. Together these findings suggest that the hNSC line we isolated are appropriately programmed and share similar characteristics with the definitive NSCs of the developing brain.

The SD56 hNSCs demonstrated a remarkable ability to migrate toward the stroke-damaged parenchyma of the adult rat brain. This directed migration by the majority of the grafted cells could be due to their uniform cellular composition, which results in an equal response to the host microenvironment.

In previous studies, subpopulations of transplanted hESCs that were enriched in neural cells migrated in host microenvironments conducive to cell migration, such as the developing brain or in structures such as the rostral migratory stream [13], [20]. In the adult lesioned brain, the grafted hESC-derived neural cells proliferated and formed rosettes [14], teratomas [12], [15] or a cellular mass that induced a gliotic host response whereby local astrocytes demarcated the grafts [16]. Enriched neural cultures derived from mouse [42] and monkey ESCs [43] have produced behavioral improvements when transplanted into animal models of stroke and brain injury. However, in these cases, the transplanted non-human ESCs also formed a mass with signs of overgrowth in the core, as well as deformations [44], [45], [46]. ESCs plated at low density acquire neural identity within few hours after plating [47]. Interestingly, nearly all viable cells expressed nestin, the early neural fate marker Sox1, and the pluripotency marker Oct4. Together, these studies are seminal and suggest that complete neuralization may not be achieved through certain enrichment processes, consequently the neural cells could revert to a pluripotent stage [17]. The dispersion of the grafted hNSCs within host parenchyma may allow for more graft-host interactions that could stabilize differentiation, inhibit growth and prevent gliotic host response.

In the present study, SD56 hNSC-transplanted animals demonstrated a stable improvement in the sensorimotor function when evaluated for spontaneous exploratory activity in the cylinder test that detects long-lasting deficits in forelimb use in the experimental models of stroke [22]. The transplantation of hNSCs significantly enhanced the independent use of the impaired contralateral forelimb 8 weeks post transplantation. This is the first report demonstrating that the transplantation of hNSCs derived from hESCs can improve neurologic behavior after experimental stroke. Together, these findings are encouraging and suggest that these cells are promising for future development. In addition to their therapeutic application, the hNSCs isolated under the reported conditions offer a means to interrogate host environments and unravel mechanistic features of self-renewal, non-tumorigenicity and functional engraftability in animal models of neurological disorders.

## Materials and Methods

### hESC and NSC Cultures

The hESC line H9 (WiCell Research Institute) was propagated every 5 to 7 days on irradiated mouse embryonic fibroblasts. The cell culture media consisted of a 1∶1 mixture of Dulbecco's modified Eagle's medium (DMEM) and F12 nutrient, 20% serum replacement (Invitrogen), 0.1 mM β-mercaptoethanol, 2 µg/ml heparin and 4 ng/ml bFGF (R&D Systems). To generate the NSCs, dissociated hESCs were cultured in a chemically defined medium composed of DMEM/F12 (1∶1) including glucose (0.6%), glutamine (2 mM), sodium bicarbonate (3 mM), and HEPES buffer (5 mM) [all from Sigma except glutamine (Invitrogen)]. A defined hormone mix and salt mixture (Sigma), including insulin (25 mg/ml), transferrin (100 mg/ml), progesterone (20 nM), putrescine (60 mM), and selenium chloride (30 nM) was used in place of serum. The medium was supplemented with EGF (20 ng/ml), bFGF (10 ng/ml) and LIF (10 ng/ml). Dissociated hNSCs were plated at a density of 100,000 cell/ml in Corning T75 (Invitrogen) culture flasks in the defined media together with the growth factors. After 5–7 DIV, the adherent culture was incubated in 0.025%trypsin/0.01% EDTA (w/v) for 1 min followed by the addition of trypsin inhibitor (Invitrogen) then gently triturated to achieve single cell suspension. The cells were then washed twice with fresh medium and reseeded in fresh growth factor-containing media at 100,000 cells/ml. This procedure was performed for 21 passages and the fold of increase and population doubling were calculated at each passage. For clonal analysis, single spheres or confluent hNSC cultures were single cell dissociated and serially diluted to yield 1–2 cell/10 µl. A 10-µl-cell suspension was then added to each of 96 or 24 well plates containing 200–300 µl of growth media. Wells containing one viable cell were marked the next day and re-scored 5 to 7 days later for cell proliferation. The differentiation of the hNSCs was performed as previously described [48]. Dissociated hNSCs were plated at a density of 10^6^ cells/ml in control (media/hormone mix) medium devoid of any growth factors and supplemented with 1% fetal bovine serum (FBS) on poly-L-ornithine-coated (15 mg/ml; Sigma) glass coverslips in 24-well Nunclon culture dishes with 0.5 ml/well. After 2, 7–15 DIV cultures were fixed and processed for immunocytochemistry or used for RT-PCR analysis.

### Karyotype analysis

Long-term cultures of hNSCs were incubated at 37°C and harvested for metaphase chromosomes when the cultures were 75% confluent. Metaphase chromosomes were obtained by standard chromosome harvest methods by exposure to Colcemid at 0.1 µg/ml for 1 hour at 37°C, a 2-minute exposure to trypsin/EDTA, hypotonized with 0.057 M KCl and fixed with 3∶1 methanol:acetic acid. Slide preparations were made by dropping the fixed cell pellet onto cold, wet slides and air-dried. After incubating the slides at 90°C for 30 minutes, chromosomes were trypsin banded and then Wright/Giemsa stained for G-banding analysis. Twenty metaphase cells were completely analyzed and a normal female chromosome complement was found (46,XX).

### Tumorigenicity in nude rats

All animal experiments were conducted according to the National Institute of Health (NIH) guidelines and approved by the IACUC. Normal adult NIH-Nude rats (n = 5, 8 week-old, Taconic, Germantown, New York, United States) were used to test the tumorigenic potential of the SD56 hNSCs. Undifferentiated hNSCs from passage 9 were single cell dissociated using trypsin-EDTA and suspended at the concentration of 125,000 cell/µl in preparation for cell transplantation. Two µl of the cell suspension were stereotaxically transplanted into 4 sites within the striatum at the following coordinates: AP: +1.0 mm, ML: +3.2 mm, DV: −5.0; AP: +0.5 mm, ML: +3.0 mm, DV: −5.0; AP: −0.5 mm, ML: +3.0 mm, DV: −5.0; AP: −1.0 mm, ML: +3.5 mm, DV: −5.0 mm with the incisor bar set at 3.4 mm. The injection rate was 1 µl/min, and the cannula was left in place for an additional 5 min before retraction. For the flank tumor assay, 2×10^6^ cells (125,000 cell/µl) were injected subcutaneously to the side of the adult nude rats. To label mitotically active cells in vivo during S-phase, the rats were injected IP with the BrdU (50 mg/kg, Sigma) every 8 hours during the last 24 hours before euthanasia. After 2-month survival time, rats were euthanized and a postmortem examination for tumor formation was performed.

### Induction of Focal Ischemia and Cell Transplantation

All animal experimentations were conducted according to the National Institute of Health (NIH) guidelines and approved by the IACUC. Sprague Dawley adult male rats (n = 17, 275g–310g, Charles River Laboratories, Wilmington, Massachusetts, United States) were subjected to one and a half hour suture occlusion of the middle cerebral artery (MCAO), as previously described [49] and immunosuppressed 2 days before cell transplantation and daily thereafter for one week with i.p. injections of cyclosporine A (20 mg/ml, Sandimmune, Novartis Pharmaceuticals). Thereafter oral cyclosporine was used at 210 µg/ml in drinking water until euthanasia. Undifferentiated SD56 hNSCs from passages between P9 and P13 were single cell dissociated using trypsin-EDTA in preparation for cell transplantation. One week after the stroke lesion, 2 µl of the hNSCs, at a concentration of 50,000 cell/µl, were stereotaxically transplanted into 4 sites within the lesioned striatum (n = 10) at the following coordinates: AP: +1.0 mm, ML: +3.2 mm, DV: −5.0; AP: +0.5 mm, ML: +3.0 mm, DV: −5.0; AP: −0.5 mm, ML: +3.0 mm, DV: −5.0; AP: −1.0 mm, ML: +3.5 mm, DV: −5.0 mm with the incisor bar set at 3.4 mm. The injection rate was 1 µl/min, and the cannula was left in place for an additional 5 min before retraction. As a control group, we used rats subjected to ischemia and injected with the vehicle (n = 7). All animals underwent baseline motor behavioral assessment before and after the ischemic lesion, and 4 & 8 weeks after cell transplantation. The animals were killed after 2-month survival time by transcardial perfusion with phosphate buffered saline (PBS) followed by 4% paraformaldehyde. The brains were cryoprotected in an increasing gradient of 10, 20 and 30% sucrose solution and cryostat sectioned at 40 µm and processed for immunocytochemistry.

### Immunocytochemistry

Cultures were fixed with 4% paraformaldehyde for 15 min. Both cells and brain sections were rinsed in PBS for 3×5 min then incubated for 2 hrs (cultures) or overnight (brain sections) with the appropriate primary antibodies for multiple labeling. Secondary antibodies raised in the appropriate hosts and conjugated to FITC, RITC, AMCA, CY3 or CY5 chromogenes (Jackson ImmunoResearch) were used. Cells and sections were counterstained with the nuclear marker 4′,6-diamidine-2′-phenylindole dihydrochloride (DAPI). Positive and negative controls were included in each run. Immunostained sections were coverslipped using fluorsave (Calbiochem) as the mounting medium. The following antibodies were used: Anti-human Nuclei (hNuc, monoclonal 1∶100, Chemicon), Anti-TuJ1 (monoclonal 1∶100, Covance; Polyclonal 1∶200, Aves Labs); anti-GAD65/67 (polyclonal 1∶1000, Chemicon); Anti-glial fibrillary acidic protein (GFAP, monoclonal 1∶1000, Chemicon; polyclonal 1∶200, Aves Labs); Anti-galactocerebrocide (GC, monoclonal 1∶250, Chemicon); Anti-CNPase (polyclonal 1∶200, Aves Labs); Anti-Glucose Transporter type 1 (Glut-1 polyclonal, 1∶500, Chemicon); Anti-Nestin (polyclonal 1∶1000, Chemicon); Anti-vimentin (monoclonal 1∶500, Calbiochem); Anti-3CB2 (monoclonal 1∶500, Developmental Studies Hybridoma Bank); Anti-doublecortin (DCX, polyclonal 1∶250, SantaCruz Biotechnology); Anti-Ki67 (polyclonal 1∶250, SantaCruz Biotechnology). Fluorescence was detected, analyzed and photographed with a Zeiss LSM550 laser scanning confocal photomicroscope. For each animal, quantitative estimates of the total number of grafted cells were stereologically determined using the optical fractionator procedure [50]. A computer-assisted image analysis system was performed using Stereo Investigator software (MicroBrightField, Inc.). The rostral and caudal limits of the reference volume were determined by first and last frontal sections containing grafted cells. The striatum and cortex were accurately outlined at low magnification (2.5× objective). The optical fractionator probe was selected to perform systematic sampling of the immunoreactive cell population distributed within the serial sections to estimate the population number in the volume of tissue. The counting frame of the optical fractionator was defined at 50×50 µm squares and the systematic sampling was performed by translating a grid with 200×200 µm squares onto the sections of interest using the Stereo Investigator software. The sample sites were systematically and automatically generated by the computer and examined using a 60× objective of a Nikon Eclipse TE 300 microscope. The counting frame displayed inclusion and exclusion lines and only immunoreactive cell bodies falling within the counting frame with no contact with the exclusion lines were counted. The cell dispersion was measured by counting the number of cells within 200 µm distance from the graft site. The number and distance in µm of cells dispersed beyond 200 µm was also measured. An average of 2,000 cells was counted per animal. Double labeling was determined using the confocal laser scanning microscope by random sampling of 100 or more cells per marker for each animal, scoring first for hNuc+, followed by DAPI+ nuclei and then the marker of choice. The double labeling was always confirmed in x-z and y-z cross-sections produced by the orthogonal projections of z-series.

### Reverse Transcription-Polymerase Chain Reaction (RT-PCR) analysis

Total RNA was extracted from cultured cells using RNAeasy kit (Quiagen). Aliquots (1 µg) of total RNA from the cells were reverse transcribed in the presence of 50 mM Tris-HCl, pH 8.3, 75 mM KCl, 3 mM MgCl2, 10 mM DTT, 0.5 µM dNTPs, and 0.5 µg oligo-dT(12–18) with 200 U Superscript RNase H-Reverse Transcriptase (Invitrogen). PCR amplification was performed using standard procedure with Taq Polymerase. Aliquots of cDNA equivalent to 50 ng of total RNA were amplified in 25 µl reactions containing 10 mM Tris-HCl, pH 8.3, 50 mM KCl, 1.5 mM MgCl2 , 50 pmol of each primer, 400 µM dNTPs, and 0.5 U AmpliTaq DNA polymerase (Perkin-Elmer). PCR was performed using the following thermal profile: 4 min at 94°C; 1 min at 94°C, 1 min at 60°C, 1.5 min at 72°C, for 30–40 cycles; 7 min at 72°C, and finally a soak at 4°C overnight. The following day, 10 µl aliquots of the amplified products were run on a 2% agarose Tris–acetate gel containing 0.5 mg/ml ethidium bromide. The products were visualized through a UV transilluminator, captured in a digital format using Quantify One Gel Analysis software (Bio-Rad Laboratories) on a Macintosh G4 computer.

The PCR primers specific to each transcript were as follows: GFAP, forward (F), 5′-TCATCGCTCAGGAGGTCCTT–3′ Reverse (R), 5′-CTG TTGCCAGAGATGGAGGTT–3′; MAP2 (F) 5′-GAAGACTCGCATCCGAATGG–3′, (R) 5′-CGCAGGATAGGAGGAAGAGACT–3′; MBP (F) 5′-TTAGCTGAATTC GCGTGTGG–3′, (R) 5′-GAGGAAGTGAATGAGCCGGTTA-3′ were deigned using the Primer Designer software, Version 2.0 (Scientific and Educational Software) [48]. 18S, β-tubulin class III, N-CAM, Nestin, NF-M, Notch-1 primers [51]. Oct4, Nanog primers [11]. FOXa2 (HNF3B), Brachyury primers [52].

### Behavioral tests

The cylinder test was used to assess the spontaneous forelimb use during lateral exploration movement [22]. Rats were placed in a transparent acrylic cylinder (20 cm diameter) for 5 minutes. The cylinder encourages use of the forelimbs for vertical exploration. A mirror was placed behind the cylinder so that the forelimbs could be viewed at all times. Testing sessions were videotaped and forelimb use was scored by a blinded operator. Movements scored were the independent use of the left or right forelimb or simultaneous use of both the left and right forelimb to contact the wall of the cylinder during a full rear, to initiate a weight-shifting movement, or to regain center of gravity while moving laterally in a vertical posture along the wall. Animals were tested for their baselines after stroke and 4 and 8 weeks after cell transplantation.

### Statistical analysis

Outcome measurement for each experiment was reported as mean±SEM. All data were analyzed using SPSS 11 for Mac OS X (SPSS Inc.). Significance of inter-group differences was performed by applying Student's t-test where appropriate. The One-Way ANOVA analysis was used to compare group differences for the forelimb use as the dependant variable and groups as the single independent factor variable. Differences between the groups were determined using Bonferroni's post hoc test. A P-value of less than 0.05 was considered to be statistically significant.

## Supporting Information

Figure S1 Schematic representation of the isolation and perpetuation processes of the SD56 hNSCs. Neural stem cells were derived from hESCs and propagated using defined media supplemented with EGF, bFGF and LIF (see Results section for details). The developmental progression of the in vitro neural specification and patterning was monitored by the expression of lineage markers as indicated at each stage.
(0.38 MB TIF)

Figure S2 Asymmetric segregation of BrdU and symmetric expression of Nestin. Dissociated hNSCs were plated at clonal density (1–2 cell/10 µl), pulsed with BrdU and immuno-processed for nestin expression by the progeny. After 5 DIV, BrdU labeling demonstrated that asymmetric segregation of the chromatids rarely occurs (arrow shows one example) in clonally derived cells. Bars: 20 µm. asymmetric segregation of the chromatids rarely occurs (arrow shows one example) in clonally derived cells. Bars: 20 µm.
(1.96 MB TIF)

Figure S3 BrdU incorporation by proliferating cells in the forebrain of naïve nude rats. Photos show frontal sections through the graft (A) and the subventricular zone (SVZ) (B) in the striatum immunostained with the human specific anti-hNuc (green in A) and anti-BrdU (red, A & B) showing 2 BrdU+ cells in graft zone (A) and the host BrdU+ endogenous SVZ transit amplifying neural precursors (B). Bars: 20 µm.
(6.76 MB TIF)
