# Supplementary figures and images for: Adherent Self-Renewable Human Embryonic Stem Cell-Derived Neural Stem Cell Line: Functional Engraftment in Experimental Stroke Model
Source: PLoS One. 2008 Feb 20;3(2):e1644. doi: 10.1371/journal.pone.0001644 (PMC2238795; doi:10.1371/journal.pone.0001644)

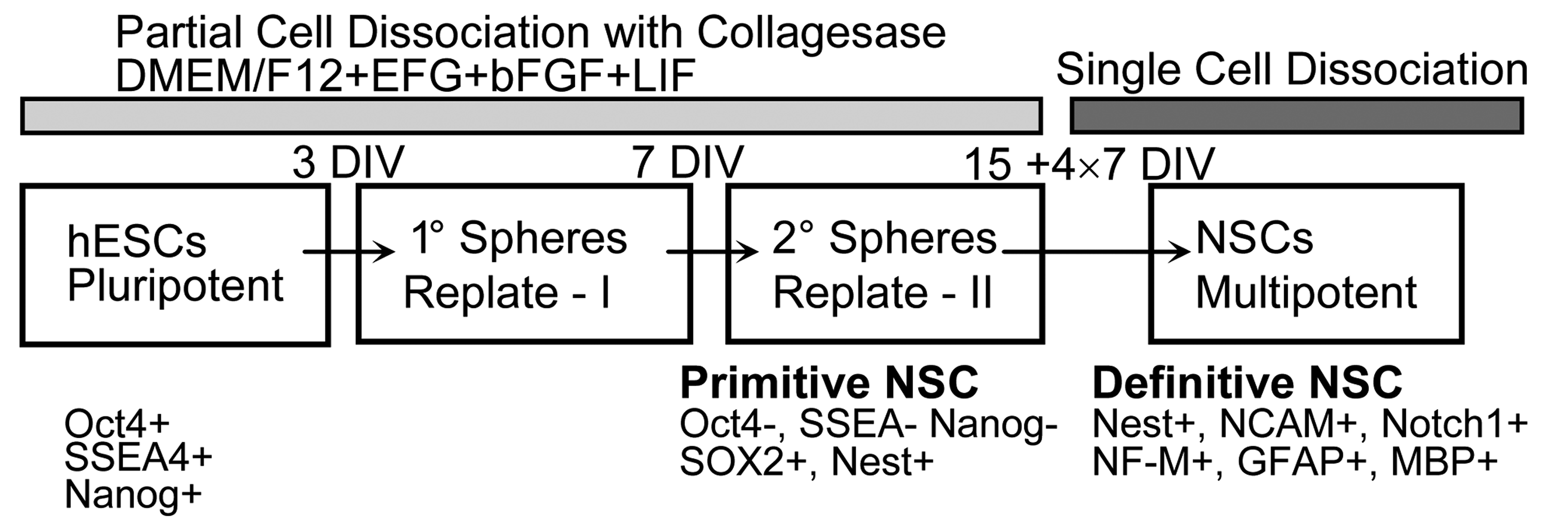

Supplement: Figure S1 — Schematic representation of the isolation and perpetuation processes of the SD56 hNSCs. Neural stem cells were derived from hESCs and propagated using defined media supplemented with EGF, bFGF and LIF (see Results section for details). The developmental progression of the in vitro neural specification and patterning was monitored by the expression of lineage markers as indicated at each stage. (0.38 MB TIF) [file pone.0001644.s001.tif]

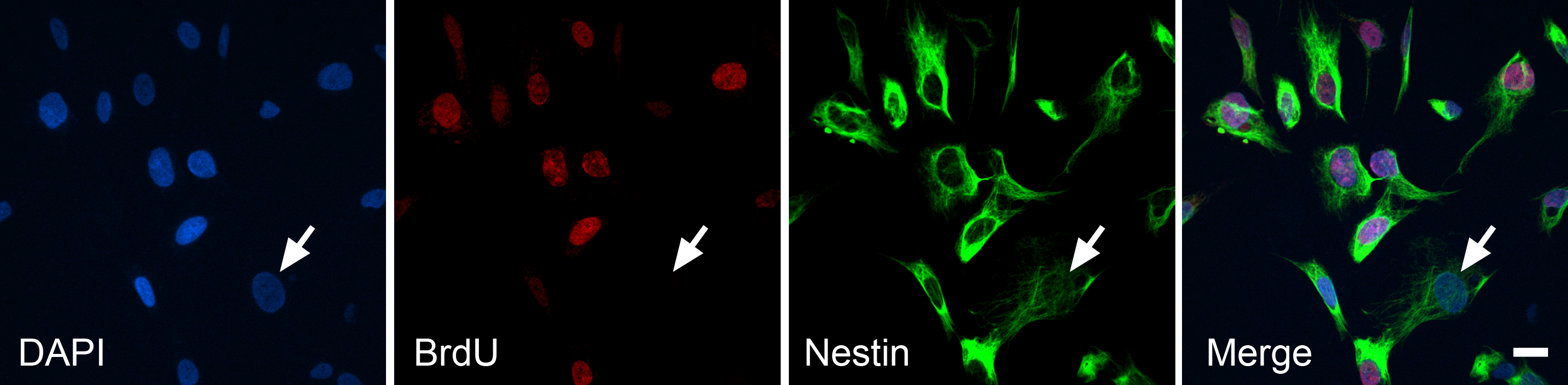

Supplement: Figure S2 — Asymmetric segregation of BrdU and symmetric expression of Nestin. Dissociated hNSCs were plated at clonal density (1–2 cell/10 µl), pulsed with BrdU and immuno-processed for nestin expression by the progeny. After 5 DIV, BrdU labeling demonstrated that asymmetric segregation of the chromatids rarely occurs (arrow shows one example) in clonally derived cells. Bars: 20 µm. asymmetric segregation of the chromatids rarely occurs (arrow shows one example) in clonally derived cells. Bars: 20 µm. (1.96 MB TIF) [file pone.0001644.s002.tif]

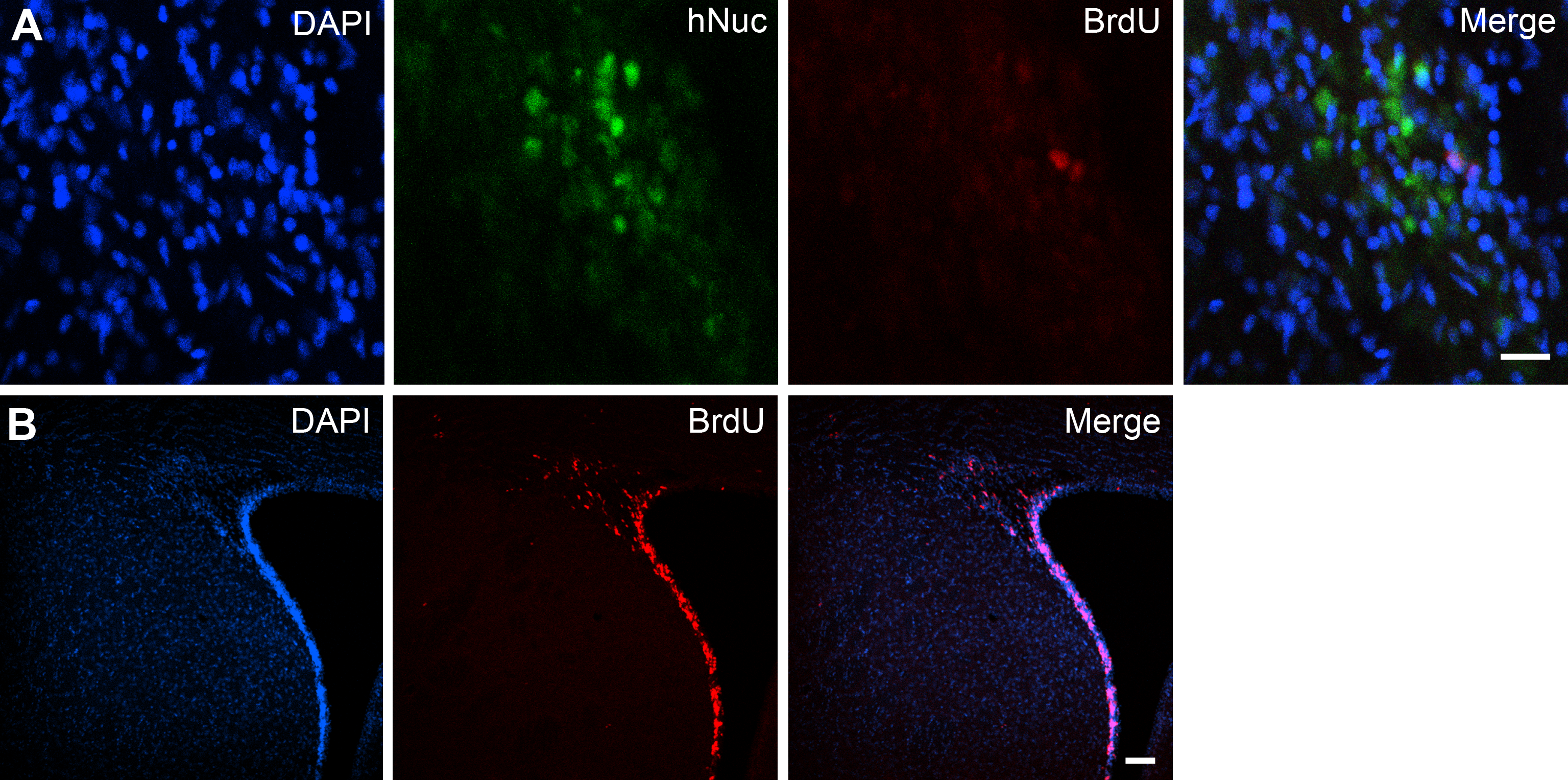

Supplement: Figure S3 — BrdU incorporation by proliferating cells in the forebrain of naïve nude rats. Photos show frontal sections through the graft (A) and the subventricular zone (SVZ) (B) in the striatum immunostained with the human specific anti-hNuc (green in A) and anti-BrdU (red, A & B) showing 2 BrdU+ cells in graft zone (A) and the host BrdU+ endogenous SVZ transit amplifying neural precursors (B). Bars: 20 µm. (6.76 MB TIF) [file pone.0001644.s003.tif]
